# Study of Oil Particle Concentration Vertical Distribution of Various Sizes under Displacement Ventilation System in Large-Space Machining Workshop

**DOI:** 10.3390/ijerph19116932

**Published:** 2022-06-06

**Authors:** Fei Wang, Qinpeng Meng, Chengjie Lin, Xin Wang, Wenbing Weng

**Affiliations:** School of Environment and Architecture, University of Shanghai for Science and Technology, Shanghai 200093, China; qinpeng_meng_usst@outlook.com (Q.M.); 18964618001@163.com (C.L.); wangxinshiyun@126.com (X.W.); wenbing@vip.163.com (W.W.)

**Keywords:** oil particle concentration, vertical distribution, large-space, displacement ventilation

## Abstract

The widespread use of metal working fluids (MWFs) in machining processes leads to the production of a large number of harmful oil particles, which may pose serious health hazards to workers. The oil particle concentration has an inhomogeneous distribution in large spaces under displacement ventilation (DV) system, and the supply air volume required to maintain a low particle concentration under a DV system may be less than that needed under a mixing ventilation system. In this study, computational fluid dynamics (CFD) was used to study the particle concentration distribution rules and characteristics under various particle sizes in a large-space machine workshop with a DV system. Several distribution indices, such as the inhomogeneity factor and stratification height were utilized to analyze the inhomogeneous distribution of particle concentration; furthermore, sensitivity analyses were conducted for these indices. We found that the particle concentration shows a similar inhomogeneity factor distribution rule along the vertical direction under an air change rate of 2–6 in the DV system. The workspace inhomogeneity factor of particles smaller than 5 μm is less than 0.25, whereas that of 10-μm particles declines with an increase in air supply volume. Approximately double the supply air volume is required to keep the 10-μm particle concentration at the same level as particles smaller than 5 μm. The workspace inhomogeneity factor of small particles (<5 μm) is more sensitive to the machine height and machine surface temperature than other parameters, whereas that of large particles (>5 μm) is more sensitive to the supply air volume than other parameters. The results of this study can be applied for the design and control of displacement ventilation systems in large-space machining workshops.

## 1. Introduction

Machinery manufacturing is a critical industry, the value of which accounts for 21.2% of China’s gross domestic product (GDP), and which is still growing at high speed [1]. Metal working fluids (MWFs) are widely used in machining processes, leading to the production of a large number of oil particles [2,3,4,5,6]. Of these particles, those with a size smaller than 10 μm can remain suspended in the indoor environment for a long time [7], These oil particles may be inhaled by people exposed to this environment and deposited in their respiratory system [8,9], causing respiratory diseases [10,11], skin diseases [12], immune system diseases [13], and even cancers [14,15].

Oil particle concentration limit advice has been provided by many national institutes due to the associated health hazards. The National Institute for Occupational Safety and Health (NIOSH) has given 0.5 mg·m^−3^ as a recommended standard, which is widely accepted [16]; however, it is difficult for machining workshops to control the oil particle concentration under this value.

Fu et al. [17] have investigated the concentration of oil particles in 43 machining workshops in East China, where only 17 of them had a concentration less than 0.5 mg m^−3^; furthermore, 6 workshops had oil concentrations greater than ten times that of the standard. Long et al. [18] have monitored the concentration of oil particles in a typical automobile parts machining workshop. The particle concentration was obviously greater than 0.5 mg·m^−3^ when the machining equipment was operating, which constituted the primary particulate source in this workshop.

**Figure 1:**
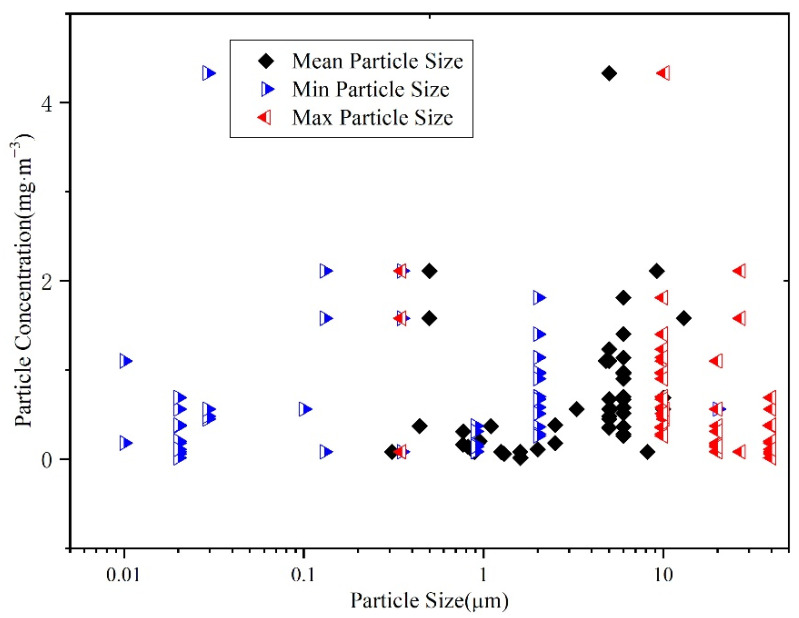
Oil particle concentration and size range in typical workshops.

Particle size characteristics have also been measured by researchers [18,19,20,21,22,23,24,25,26,27,28,29,30]. Figure 1 shows the results for 50 typical factories, including fastener processing factories [19,20], charging machinery processing factories [21], and machining factories [22,23,24,25,26,27,28,29]. The blue triangles in the figure represent the minimum particle size, whereas the red triangles and black rhombuses represent the maximum, and mean particle size, respectively. The *y*-axis represents the mass concentration of oil mist particles in the factories. It can be seen that the particle sizes in factories are typically less than 30 μm, and the mean particle sizes are between 0.3 and 10 μm. The most common particle sizes in the factories are between 2 and 10 μm.

Ventilation is still the most effective technical method for reducing particle concentration. Setting local exhaust hoods near the emission source is a popular solution. However, due to installation position restrictions and air disturbances caused by the loading and unloading of workpieces, these exhaust hoods cannot capture 100% of the oil particles. Therefore, an overall ventilation system, based on the dilution principle, is still necessary for workshop particle concentration reduction.

Jiao et al. [31] have compared the CO removal efficiency of seven types of ventilation air distribution system in industrial plants by using computational fluid dynamics (CFD) methods. Their results showed that the air pattern has a great influence on the contaminant removal efficiency.

Through CFD simulation, Feigley et al. [32] have found that an inhomogeneous particle concentration distribution will increase the risk of people exposed to high-concentration particles. They proposed a dilution safety factor for correcting the ventilation volume calculation when the risk needs to be decreased. Therefore, an inhomogeneous particle concentration will change the required ventilation volume. Machining workshops are usually large-space buildings, of which only no more than 30% in the lower part is an occupation zone. Therefore, the inhomogeneous distribution of particles in this space is more important than that of a normal room (e.g., office, apartment).

Displacement ventilation (DV) systems, which can take advantage of thermal and pollution concentration stratification, have been widely considered by researchers in order to control the temperature or pollutant concentration in the occupation zone. Wang et al. [33] have studied the influence of the supply air vent height on welding fume transfer and the particle concentration in the breathing zone of a welding factory through experiments and CFD simulation, and obtained the best supply air vent height.

Zhang et al. [34] have compared the performance of three ventilation systems in a large-space machining workshop by using CFD methods: a roof exhaust system, a combined roof exhaust and air re-circulation system, and a combined roof exhaust and displacement ventilation system. Their results indicated that the combined roof exhaust and displacement ventilation system is the most suitable for the large-space workshop. 

Meanwhile, many improved displacement ventilation systems have been developed. Wei et al. [35] have designed a novel ventilation system to solve this problem, which includes a cylindrical downward air supply vent and an infrared induction device. Their CFD simulation results demonstrated that the novel ventilation system can reduce the concentration of oil particles in the occupation zone by 70–76%, under the condition of meeting thermal comfort needs in the workshop in winter and summer.

Wang et al. [36] have created a vortex airflow by adjusting the supply air angle of the DV system to improve the contaminant removal efficiency. Their CFD results showed that the contaminant removal efficiency of the vortex DV system is higher than that of an ordinary DV system, but the contaminant source position has a significant influence on the removal efficiency.

It can be seen that the advantages of DV systems, which can be applied in the machining workshops, have been proved by many researchers. However, there have been few studies focused on how to correct the supply air volume of the DV system to reduce the oil particle concentration when considering an inhomogeneous distribution, as the rules of inhomogeneous distributions of oil particles in large-spaces remain unclear.

Wang et al. [37] have studied the particle concentration distribution in the vertical direction by using a CFD method, and observed stratification of the particle concentration along vertical direction, however, they did not further analyze and discuss the influencing factors and rules of this particle concentration stratification. Therefore, it is both important and urgent to study the particle concentration distribution rules.

In summary, MWF particles are harmful to the people who work in machining workshops. Furthermore, DV systems have better ventilation efficiency for the occupied zone in large-space workshops, compared to other ventilation forms (e.g., mixing ventilation), due to the contamination stratification phenomenon. However, the existing literature has neither provided a conclusion regarding the stratification and inhomogeneity rules of the oil particle concentration, nor studied the supply air volume correction method for the DV systems to reduce the oil particle concentration under various particle sizes, when inhomogeneous distribution is considered. Therefore, it is very meaningful to study the distribution rule for the concentrations of various particle sizes along the vertical direction under a DV system.

## 2. Methodology

### 2.1. Numerical Models and Solver Setting

In this paper, CFD methods were employed to simulate indoor airflow, air temperature, and oil particle concentration. The Reynolds average Navier–Stokes (RANS) approach has been successfully validated for the prediction of airflows and, they were consequently employed in this study. The governing equations [38] are as follows:

(1) $$\frac{\partial u_{j}}{\partial x_{j}}=0,$$

(2) $$\frac{\partial \left(u_{j}u_{i}\right)}{\partial x_{j}}=\frac{\partial }{\partial x_{j}}\left(\left(\mu +\mu_{t}\right)\frac{\partial u_{i}}{\partial x_{j}}\right)-\frac{1}{\rho }\frac{\partial P}{\partial x_{i}}+g_{i},$$

(3) $$\frac{\partial \left(u_{j}T\right)}{\partial x_{j}}=\frac{\partial }{\partial x_{j}}\left(\left(\frac{\mu }{P_{r}}+\frac{\mu_{t}}{\sigma_{t}}\right)\frac{\partial T}{\partial x_{j}}\right)+S_{T},$$

where $u_{j}$ is the velocity component (m·s^−1^), $u_{i}$ is the time-averaged velocity (m·s^−1^), μ is the molecular viscosity (Pa·s), $\mu_{t}$ is the turbulent viscosity (Pa·s), P is the pressure (Pa), $g_{i}$ is the gravitational body force (m·s^−2^), ρ is the density (kg·m^−3^), and T is the temperature (K).

As Zhang et al. [34] and Wei et al. [35] have shown, the renormalization group RNG k-ε turbulence model has good performance in simulating the air velocity field, air temperature field, and oil particle concentration field in industrial factories, as the simulation results were in good agreement with spot-measured data. Therefore, the RNG k-ε model was also used in this paper. The transport equations for the turbulent kinetic energy *k* and turbulence dissipation rate ε are as follows:

(4) $$\frac{\partial k}{\partial t}+\frac{\partial \left(ku_{j}\right)}{\partial x_{j}}=\frac{\partial }{\partial x_{j}}\left(\frac{v_{t}}{\sigma_{k}}\frac{\partial k}{\partial x_{j}}\right)+P_{k}-\varepsilon ,$$

(5) $$\frac{\partial \varepsilon }{\partial t}+\frac{\partial \left(\varepsilon u_{j}\right)}{\partial x_{j}}=\frac{\partial }{\partial x_{j}}\left(\frac{v_{t}}{\sigma_{\varepsilon }}\frac{\partial \varepsilon }{\partial x_{j}}\right)+\frac{\varepsilon }{k}\left(C_{\varepsilon 1}^{*}P_{k}-C_{\varepsilon 2}^{*}\varepsilon \right),$$

where Pr is the Prandtl number, $\sigma_{t}$ is the turbulent Prandtl number (also written as $Pr_{t}\;$= 0.85), $S_{T}$ is the heat source term (W·(m^3^·s)^−1^), *k* is the turbulent kinetic energy, ε is the turbulence dissipation, $v_{t}$ is the kinematic eddy viscosity ($v_{t}=C_{\mu }\frac{k^{2}}{\varepsilon }$), $\sigma_{k}$ = 0.7194, $P_{k}$ = $v_{t}$*S*^2^, $S=\sqrt{2S_{ij}S_{ij}}$, $S_{ij}=\frac{1}{2}\left(\frac{\partial u_{i}}{\partial x_{j}}+\frac{\partial u_{j}}{\partial x_{i}}\right)$, $C_{\varepsilon 1}^{*}$ = 1.42 − $\frac{\eta \left(1-\eta /4.38\right)}{1+0.012\eta^{3}}$, $\eta =\frac{k}{\varepsilon }S$, $C_{\varepsilon 2}^{*}$ = 1.68, and $\sigma_{\varepsilon }$ = 0.7179.

There are many optional models for calculating the particulate matter concentration. Previous studies have shown that the Lagrange method with the discrete random walk (DRW) model has the best fit to experimental data for indoor particle concentration computation [33,39].

The basic idea of the Lagrange method is to track the trajectories of particles, and then convert the trajectories into the concentration of particles. The governing equation of a particle’s motion is based on Newton’s law of momentum [40]:

(6) $$\begin{array}{c} \frac{d{\vec{u}}_{p}}{dt}={\vec{F}}_{drag}+\vec{g}\left(\frac{\rho_{p}-\rho_{a}}{\rho_{a}}\right)+{\vec{F}}_{x}, \end{array}$$

where ${\vec{u}}_{p}$ is the velocity vector of particles in the air, ${\vec{F}}_{drag}$ is the drag force vector of particles in the air, $\rho_{p}$ and $\rho_{a}$ are the density of particles and the density of air, respectively; $\vec{g}$ is the acceleration due to gravity, and ${\vec{F}}_{x}$ denotes the other additional forces.

If the Reynolds number is small (Re < 1), the particle is in the Stokes region, and the drag force conforms to Equation (7):

(7) $$\begin{array}{c} {\vec{F}}_{drag}={\vec{F}}_{D}\left({\vec{u}}_{a}-{\vec{u}}_{p}\right)=\frac{18\mu }{\rho_{p}D_{p}^{2}C_{c}}\left({\vec{u}}_{a}-{\vec{u}}_{p}\right), \end{array}$$

where μ is the dynamic viscosity of air, $D_{p}$ is the particle diameter, and $C_{c}$ is the Cunningham factor: $1+\frac{2\lambda }{D_{p}}\left(1.257+0.4e^{-\left(\frac{1.1D_{p}}{2\lambda }\right)}\right)$.

The other additional forces include the Basset force, pressure gradient force, virtual mass force, Brownian force, thermophoresis force, Saffman force, and so on. Tian et al. [41] have conducted an order-of-magnitude analysis of the above forces, and found that the drag force and gravity were still the dominant forces, whereas the other forces were two orders of magnitude smaller than these two forces. Li et al. and other researchers [42,43,44] have found that some of the additional forces suddenly increase to the same order of magnitude as the drag force in the turbulent boundary layer, which may affect the deposition computation. However, in a large-space building, natural convention dominates the air flow of boundary, and deposition at the wall has little influence on the indoor particle concentration distribution; thus, gravity and drag forces were mainly considered in this paper.

The Lagrange method can only track the trajectories of particles. Therefore, the PSI-C algorithm was used to transform the particles trajectories into particle concentrations. This algorithm has been proposed and validated by Zhang [45]. When processing particle tracking, the influence of particle motion on continuous terms was ignored. Furthermore, as the oil particles are liquid, they were assumed to be captured when they reached the wall.

The Fluent module of Ansys17.0 was employed to solve the temperature field, velocity field, and particle concentration field. A standard wall function was selected for the near-wall treatment. The pressure parameter adopted the staggered grid PRESTO! method, and other parameters (e.g., the momentum) were discretized by the second-order upwind method. The SIMPLE algorithm was used to decouple the pressure and velocity [39,46]. Default options and constants in the Fluent software were applied for the other settings of the turbulence model and solver.

According to previous studies, air was considered to be an incompressible fluid, and the Boussinesq hypothesis was adopted [47,48,49,50].

The criteria of convergence mainly included several aspects: The energy calculation residual was less than 1.0 × 10^−6^, while other parameters were less than 1.0 × 10^−3^; and the flow balance and heat balance of the whole space were less than 1%. The parameter values at the concerned points tended to be stable. 

The selection, setting, and input information for the used CFD models are provided in Table 1.

### 2.2. CFD Validation

**Figure 2:**
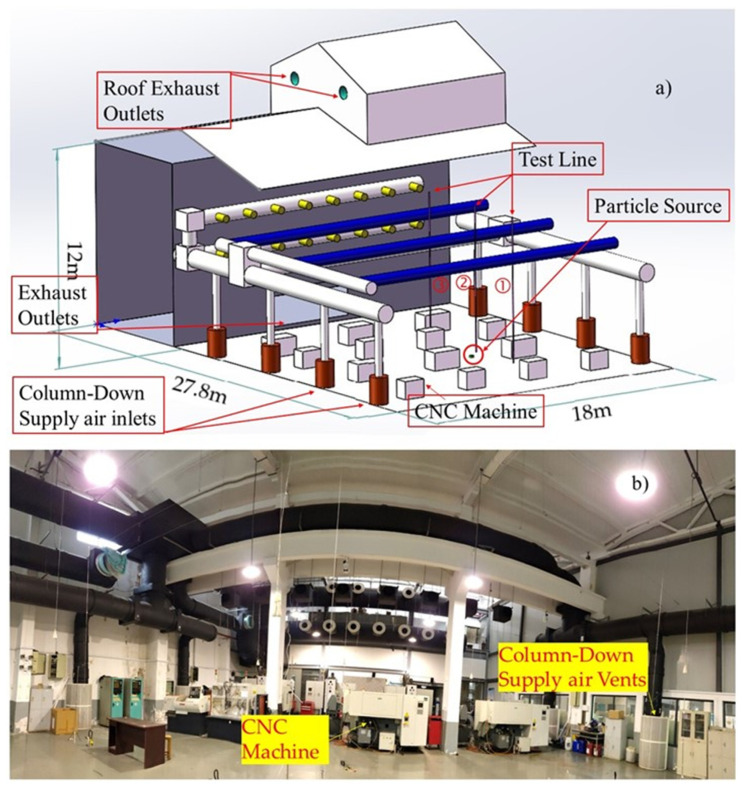
Experimental facility and ventilation system for CFD validation: (**a**) Diagram of experimental facility, and (**b**) picture of experimental indoor scene.

In order to validate the CFD model, an experiment was conducted in a large-space building, which is used as a central navigation computer (CNC) machine training center. As shown in Figure 2, the experimental area was 27.8 m long, 18 m wide, and the highest part of the roof was 12 m over the floor. The volume of the whole space was 6048 m³. The ventilation system used in the experiment included eight column-down supply air vents along two parallel walls, an exhaust outlet on the lower part of the wall, and two exhaust fans on the roof part. The emission rate at the particle source was tested by using equipment designed according to ISO 5801:2007 [51]. Others detailed information and the CFD boundary conditions can be found in the Supplementary Materials (see Section S1).

**Figure 3:**
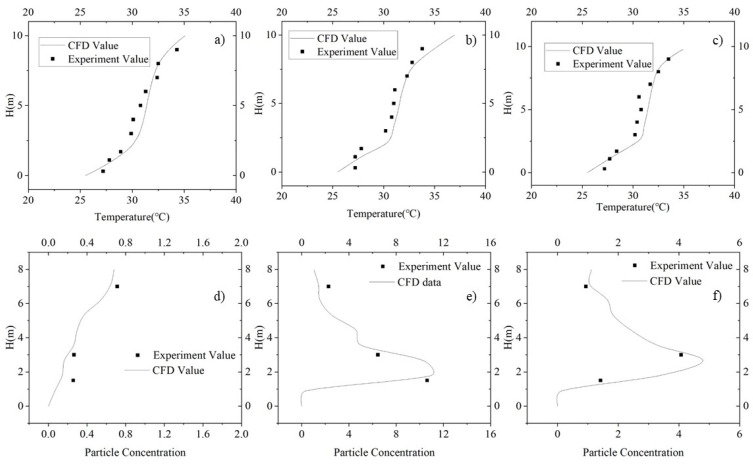
Comparison of CFD and experimental temperature and particle concentration data: (**a**) CFD and experimental temperature data for test line 1, (**b**) CFD and experimental temperature data for test line 2, (**c**) CFD and experimental temperature data for test line 3, (**d**) CFD and experimental particle concentration data for test line 1, (**e**) CFD and experimental particle concentration data for line 2, and (**f**) CFD and experimental particle concentration data for line 3.

The CFD simulation and experimental results are shown in Figure 3. It can be seen, from Figure 3a–c, that the difference between the simulated temperatures and experiment temperature of lines 1–3 is less than 1 °C. Figure 3d–f shows the particle concentrations for the simulated and experimental data. The particle concentration data were normalized by using

(8) $$\begin{array}{c} C_{i\_g}=\frac{C_{i\_indoor}-C_{i\_s}}{\frac{E_{i}}{Q_{sa}}},\;\;\;\; \end{array}$$

where $C_{i\_g}$ is the normalized particle concentration of size *i*, $C_{i\_indoor}$ is the indoor particle concentration of size *i* (mg·m^−3^), $C_{i\_s}$ is the supply air particle concentration of size *i* (mg·m^−3^), $Q_{sa}$ is the supply air volume (m^−3^·s^−1^), and $E_{i}$ is the particle emission rate of size *i* (mg·s^−1^). The emission rate test method is detailed in the Supplementary Materials (see Section S1). It can be seen, from Table S2 (in the Supplementary Materials), that the particle source used in the CFD validation experiment emitted large amounts of 1.0 μm and 2.5 μm particles. In terms of particle number, the emission rate of 1.0 μm particles was approximately 8 times that of 2.5 μm particles. Furthermore, the particle detector used in the validation experiment had good accuracy (±10%) with respect to this particle size range. Thus, the concentration of 1.0 μm particles was considered more suitable than other sizes for validating the CFD results, and so, 1.0 μm particles were selected for validation. It can be seen that the CFD data for particle concentration are close to the experimental data on all test lines, except for the lowest point on line 1. The possible reason for this may be that this point in upstream of the particle source. Therefore, the particle concentration at this point was low and apt to be influenced by particle increase caused by the air supply.

According to the comparison, it can be confirmed that the selected turbulence model was in good agreement with the actual situation. The Lagrange method and the PSI-C algorithm [39] can accurately predict the particle concentration field. Therefore, for the rest of the CFD simulation, we adopted the same settings, methods, and parameters.

### 2.3. Particle Concentration Inhomogeneity and Distribution Indices

To quantitatively analyze the inhomogeneous particle concentration distribution, several indices are proposed. The inhomogeneity factor defined in Equation (9), can describe the relative distribution of particles emitted by the indoor particle sources:

(9) $$\begin{array}{c} \alpha_{i}=\frac{C_{i\_indoor}-C_{i\_s}}{\left(\frac{E_{i}}{Q_{sa}}\right)}, \end{array}$$

where $\alpha_{i}$ is the (dimensionless) inhomogeneity factor of particles of size *i*, $C_{i\_indoor}$ is the spatial concentration of particles of size *i* (mg·m^−3^), $C_{i\_s}$ is the concentration of particles of size *i* in the supply air (mg·m^−3^), $E_{i}$ is the source emission rate of particles of size *i* (mg·h^−1^), $Q_{sa}$ is the supply air volume (m^3^·h^−1^), and $\frac{E_{i}}{Q_{sa}}$ is the instantaneous homogeneously diffused concentration.

Vertical average inhomogeneity of the particle concentration is essential for ventilation system design and ventilation system control. Other indices related to the average vertical particle distribution were also considered, such as the vertical distribution centroid, vertical diffusion radius, plane diffusion radius [52,53,54,55], and stratification height.

The concept of concentration distribution moment, defined by Sandberg [53], was also used. The first-order moment of the distribution of the vertical average concentration is defined as the vertical distribution centroid, calculated as follows:

(10) $$\begin{array}{c} h_{G}=\int \frac{hC\left(h\right)}{C_{0}}d\left(h\right), \end{array}$$

where $h_{G}$ is the height of the concentration distribution center (m), h is the vertical height (m), C(h) is the vertical average concentration (kg·m^−^³), and $C_{0}$ is the spatial integral concentration (kg).

Murakami [52] has defined the second-order moment of the concentration distribution as scale for ventilation efficiency 2 (SVE2), which denotes the pollution diffusion radius. In this paper, it is used in the vertical direction, in order to indicate the vertical diffusion radius, as shown in Equation (11):

(11) $$\begin{array}{c} R_{h}^{2}=\int \frac{{\left(h-h_{G}\right)}^{2}C\left(h\right)}{C_{0}}d\left(h\right), \end{array}$$

where $R_{h}$ is the vertical direct diffusion radius (m), $h_{G}$ is the particle concentration distribution centroid height (m), h is the vertical height (m), C(h) is the average particle concentration at height *h* (kg·m^−^³), and $C_{0}$ is the spatial integral particle mass (kg).

According to the definition of $R_{h}$, particles with mass within the range of [$h_{G}-R_{h}$,$h_{G}+R_{h}$] share 68% of the total space particle mass. Thus, particles with mass within the range [0,$\;h_{G}-R_{h}$] share 16% of the total space particle mass. The stratification height is defined, based on this principle, as:

(12) $$\begin{array}{c} h_{s}=h_{G}-R_{h}. \end{array}$$

In order to explain the reason for stratification in the particle distribution, the horizontal diffusion radius is proposed in this paper. Based on the second-order moment of particle concentration distribution [52], the horizontal diffusion radius is defined as Equation (13). This index reflects the relative diffusion range in each plane at various heights:

(13) $$\begin{array}{c} R_{xy}^{2}=\int \frac{{\left[\left(x,y\right)-{\left(x,y\right)}_{s}\right]}^{2}C\left(x,y\right)}{C_{avg}*A}dz, \end{array}$$

where ${\left(x,y\right)}_{s}$ are the coordinates of the emission center (*x, y*), C(x,y) is the particle concentration at point (*x, y*) (kg·m^−^³), $C_{avg}$ is the spatial average concentration (kg·m^−^³), and *A* is the cross-sectional area (m^2^).

### 2.4. Physical Model, Grid and Boundary Condition

**Figure 4:**
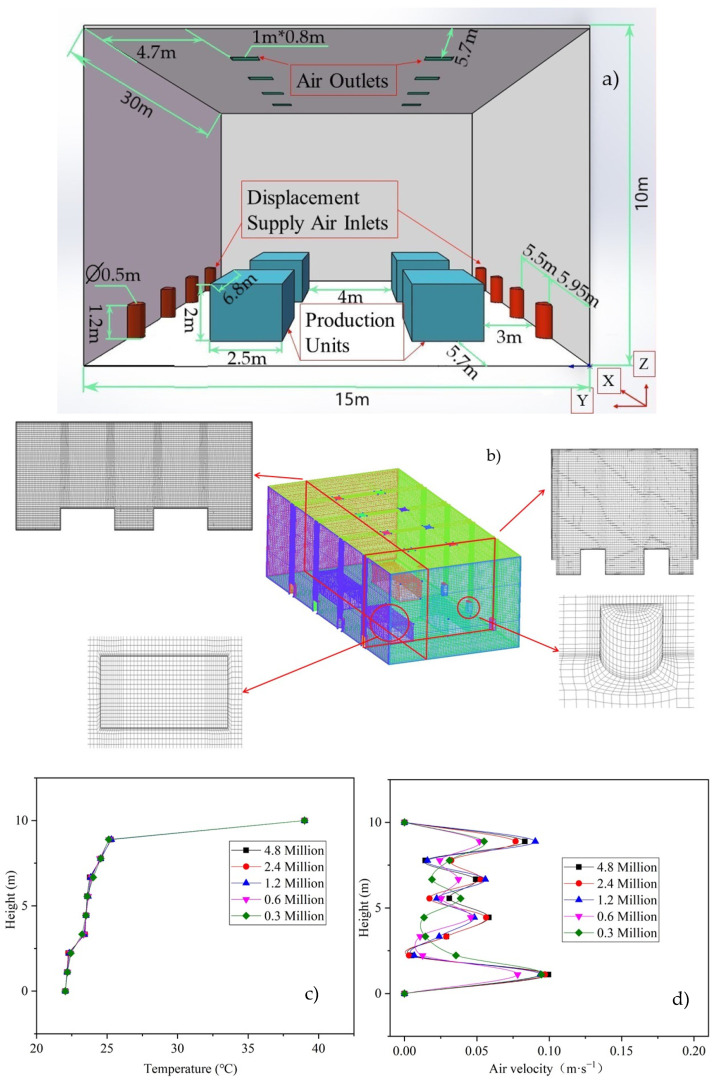
Geometric model, computational mesh, grid refinement information, and grid-independent verification results. (**a**) Geometric model used in this study, (**b**) computational mesh and grid refinement information, (**c**) temperature result of grid-independent verification, and (**d**) air velocity result of grid-independent verification.

Wang et al. [56] have investigated the indoor air quality, ventilation system, building geometric structure, and equipment layout of eight typical machining workshops. Referring to the data and results of their investigation, we simplified the large-space machining workshop to the model shown in Figure 4. The overall dimensions of the workshop are 30 m × 15 m×10 m (length × width × height). There are four production units in the workshop, each with a size of 6.8 m × 2.5 m × 2 m (length × width × height). The geometric model is symmetrical; dimensions and position information are shown in Figure 4a. 

There are eight columnar displacement air supply vents on both sides of the workshop, with height of 1.2 m and diameter of 0.5 m. The air supply vents are divided into four groups, with each group corresponding to a production unit. Eight air outlets are set on the roof, with size of 0.8 m × 1 m.

The use of high-quality grids is important to ensure that the CFD results. In this study, the ICEM software was used to generate structured grids. Grid refinement was performed in the areas where the air outlet vent, production units, and air supply vent are located. In order to ensure that the heat is sufficiently dissipated from the wall and production units into the room and wall function works properly, the grid was also refined near the wall, roof, and floor, as shown in Figure 4b. The grid size of first layer near the production unit surface and wall surface was set to 20 mm, in order to ensure that Y+ was between 30 and 150 [57,58], and the standard wall function was applied at these positions.

Five sets of different grid resolutions under the same boundary conditions were employed, in order to verify the grid-independent solution. Comparisons of the vertical temperature and air velocity distributions for a test line (X = 20.9 m, Y = 7.5 m, Z = 0–10 m) are shown in Figure 4c and Figure 4d, respectively. It can be seen that vertical temperatures of the test lines obtained with different grid simulations are similar. The air velocities obtained when using the 1.2, 2.4, and 4.8 million grids are also close. In order to compromise between the accuracy and computational time, all of the following simulations were performed by using 1.2 million grids, in which the largest grid size was less than 200 mm × 200 mm.

The CFD boundary conditions, such as the wall temperature and machine surface temperature, referred to the parameters measured by Zhang [34] in an automotive parts factory.

According to Zhang et al. [22], the major component of oil particles is mineral oils. Therefore, fuel-oil-liquid was selected as the particle material, which has a similar density to mineral oils, in the CFD software, and the particle character was set to inert. According to the literature review in this paper and our previous studies [56,59,60], the sizes of oil particles formed by machining basically range between 0.3–10.0 μm, covering two main particle size segments: fine particles (0.1~2.5 μm) and coarse particles (2.5~10 μm). Two typical particle sizes in both segment were selected as the oil particles emitted due to machining, and each particle size was studied respectively. The data processing tools of the CFD software were used to obtain the oil particle concentration directly. As the inhomogeneity factor is a dimensionless parameter, the particle emission rate was set to 1 × 10^−6^ kg·s^−1^ for convenience of data processing. Details of the boundary conditions are provided in Table 2.

## 3. Result

### 3.1. Velocity Field and Vertical Particle Concentration Distribution

**Figure 5:**
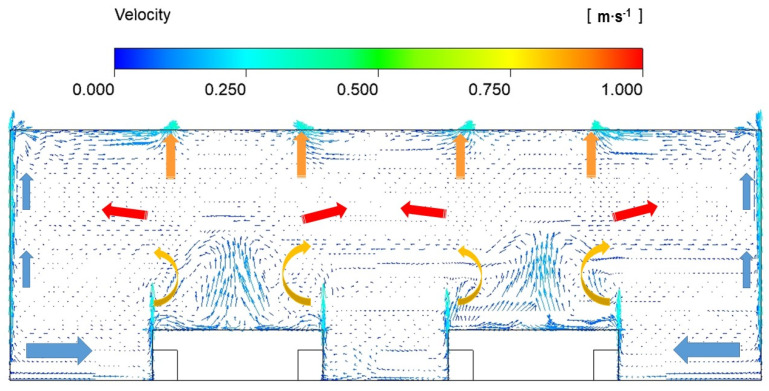
Velocity field in XZ plane at Y = 4.5 m under ACR of 3.

According to Tian [41], drag force, which is caused by the relative velocity between a particle and that of the surrounding air, is one of the major forces dominating particle motion. Thus, the particle concentration is greatly affected by the airflow distribution. The air velocity field in the XZ plane at Y = 4.5 m under an air change rate (ACR) of 3 is shown in Figure 5. It can be seen that velocity field is dominated by the supply airflow at the lower part of the space, and by the heat source plume generated by the production units, wall plume, and exhaust air at the top of the space. The heat plume cannot be connected to the exhaust airflow as the heat flux density of the machines is not strong enough. Therefore, there is a horizontal airflow layer in the middle part of the space. The air volume entrained by the heat plume is filled by supply air, which keeps attaching to the ground.

**Figure 6:**
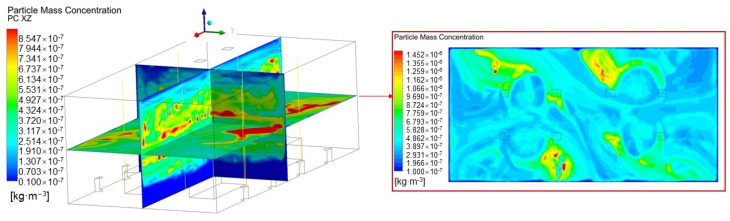
Particle concentration field in XZ plane at Y = 7.5 m, YZ plane at X = 15 m, and XY plane at Z = 5 m under ACR = 3.

The concentration distribution of 1 μm particles under an air change rate (ACR) of 3 is shown in Figure 6. It can be seen that heat plume of the machines carries the particles to the middle part of the space, causing a high particle concentration in this area. Part of the clean air supplied through the DV vents fills the lower part of the space, which creates a low particle concentration in there, whereas the other clean air reaches the top of the space along the walls, leading to a relatively low particle concentration at the top of the space.

From the particle field in the XY plane, it can be seen that the particle concentration distribution has no regular rule, and the high concentration areas seem to be distributed randomly.

**Figure 7:**
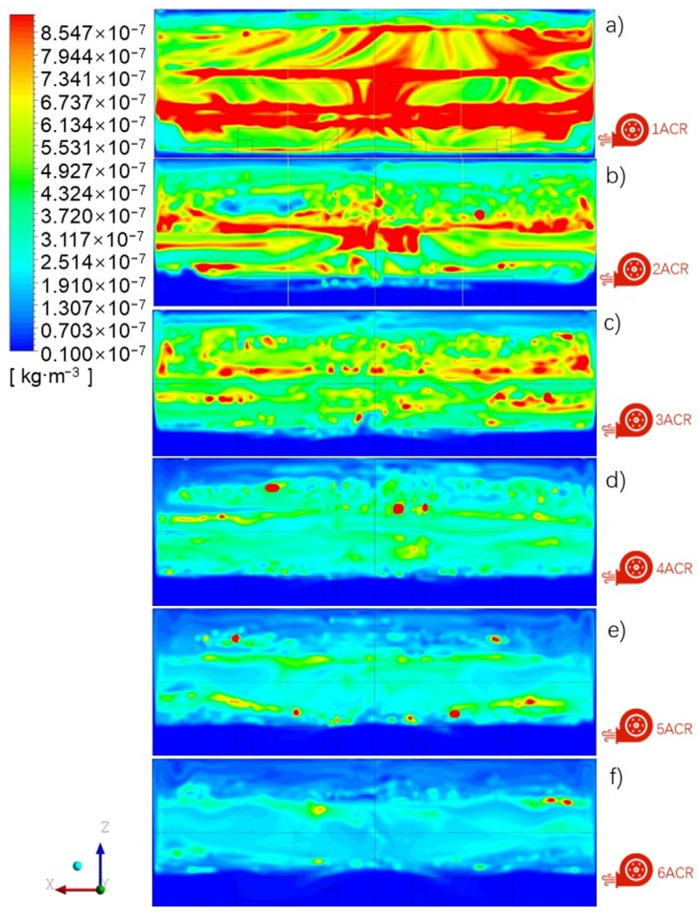
Particle concentration distribution in the XZ plane at Y = 7.5 m under various air change rate: (**a**) ACR = 1, (**b**) ACR = 2, (**c**) ACR = 3, (**d**) ACR = 4, (**e**) ACR = 5, (**f**) ACR = 6.

The supply air volume can affect the particle concentration distribution. Figure 7 shows the 1 μm particle concentration distribution in the middle XZ plane under various ACR. It can be seen that the particle concentration distribution is generally high in the middle part and low around the ground and roof under an ACR in the range of 1–6, especially with ACR greater than 2. There is an obvious stratification of particle concentration in the vertical direction, except under an ACR of 1, and the stratification height is about 2 m, which increases along with the air change rate. Meanwhile, the high concentration area is gradually compressed with an increase in supply air volume.

**Figure 8:**
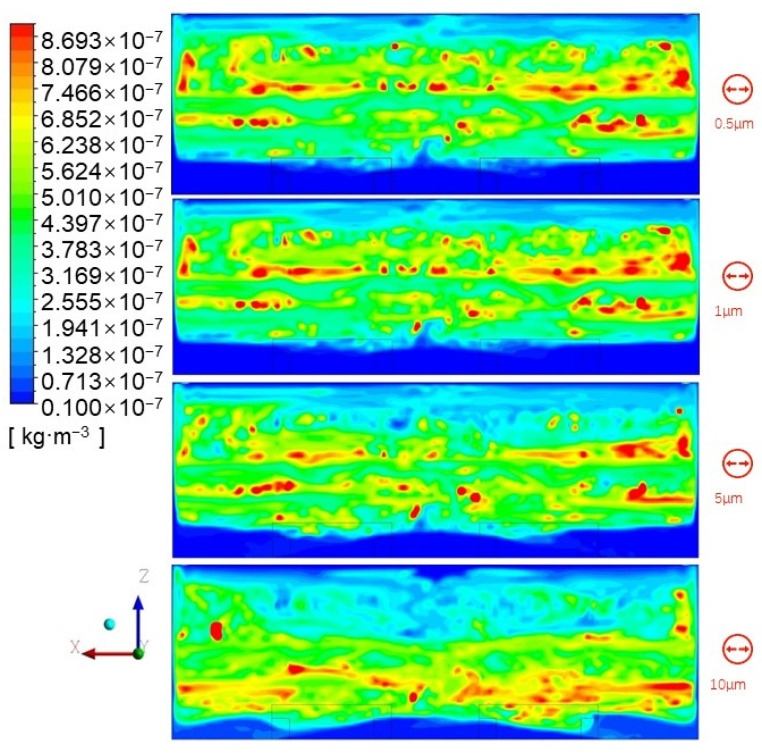
Concentration distribution of particles with various sizes in XZ plane at Y = 7.5 m under ACR = 3.

The particle concentration presented different distributions in the vertical direction due to gravity effect, in terms of various particle sizes. Figure 8 shows the distributions for four particle sizes (i.e., 0.5 μm, 1 μm, 5 μm, and 10 μm) under an ACR of 3.

It can be seen that the vertical particle concentration distributions of 0.5 μm and 1.0 μm particles do not present any significant differences. However, the 5 μm and 10 μm particle concentration distributions show considerable differences. The height of the high-concentration area of 5 μm particles and the stratification height are obviously lower than those of 0.5 μm and 1.0 μm particles. When the particle size reaches 10 μm, the high-concentration area moves further down, the stratification height decreases significantly, and the volume of the area is compressed.

It can be inferred, from Figure 8, that gravity causes large particles (>5 μm) to remain at a lower level than small particles (<5 μm). The larger the particle size, the lower the particles are distributed. Therefore, different solutions should be employed to reduce the particle concentration, when considering different particle sizes.

Oil particle concentration of other vertical planes follow the same rules. Results are not shown in this paper, for the sake of space.

### 3.2. Vertical Inhomogeneity Factor of Particle Concentration Distribution 

Figure 7 and Figure 8 show the particle concentration fields in vertical planes. However, the use of a numerical index is more suitable for quantitative studies. The inhomogeneity factor can describe the relative concentration distribution of particles. If the inhomogeneity factor is equal to 1, the mean particle concentration in an area is equal to the theoretical homogeneous mixed particle concentration, and the average inhomogeneity factor for XY planes at various heights can be used to present the relative particle concentration distribution in the vertical direction.

**Figure 9:**
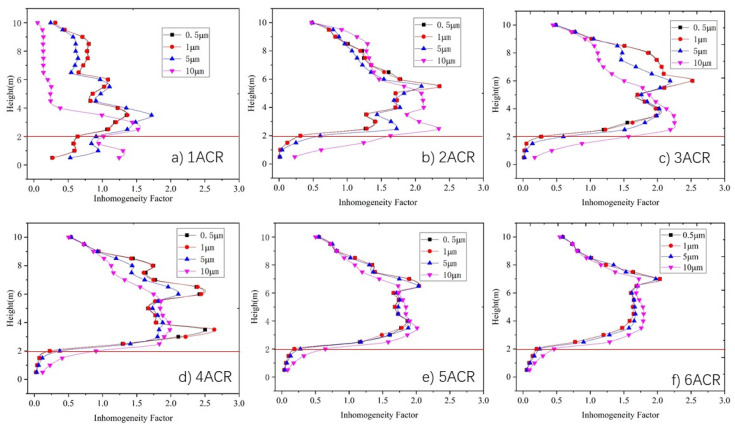
Relationship between vertical average inhomogeneity factor for particles of various sizes under different air change rate: (**a**) ACR = 1, (**b**) ACR = 2, (**c**) ACR = 3, (**d**) ACR = 4, (**e**) ACR = 5, (**f**) ACR = 6.

Figure 9 shows the average vertical inhomogeneity factor of the particle concentration distribution under an ACR ranging between 1 and 6. As Figure 9a shows, the inhomogeneity factor of particles with a size in the range of 0.5–5 μm is generally between 0.5 and 1.5 in most areas under an ACR of 1. This means that there is no clear stratification in the vertical direction, in agreement with Figure 7a. As there is insufficient supply air, the heat plume generated by the machine and the entrained airflow thoroughly mix the particles. For 10 μm particles, the velocity of the heat plume is not high enough to drag the particles to a high level, and so, they remain distributed in the lower part of the space.

When the ACR increases in the range of 2–4, as shown in Figure 7b–d, the inhomogeneity factors at 3–8m increase to 1.5–2.5, whereas those at the other heights decrease to 0–1.5. This means that most particles generated from machines accumulate in the middle height range of the space, in agreement with the results in Section 3.1. With the further increase in the air change rate (ACR) to 5–6, the inhomogeneity factors at 3–8 m decrease to 0.2–0.4.

The inhomogeneity factor is influenced not only by supply air volume, but also by the particle size. It can be seen, from Figure 9, that the inhomogeneity factor of 10 μm particles below 6 m height is greater than that of other sizes, and is less than that of the other sizes when over 6 m. The inhomogeneity factor of 5 μm particles show the similar trend, but the difference in the inhomogeneity factor between 5 μm particles and 1.0 μm particles is smaller than that between 10 μm particles and 5.0 μm, whereas the inhomogeneity factors of 0.5 μm particles and 1.0 μm seem to be exactly the same. Furthermore, the difference in the inhomogeneity factor among the four particle sizes becomes small when the supply air volume is increased.

In summary, according to Figure 9, particles begin to accumulate between a height of 3 m and 8 m when the air change rate (ACR) is greater than 2, where the particle concentration below 3 m is significantly lower than that at middle height, and the differences in particle concentration distribution among the various sizes become smaller with increased supply air volume.

In a large-space machine workshop, the operators always remain in the area below 2 m in height. Therefore, the particle concentration below 2 m is essential for the health of the people who work in the workshop, as well as the particle concentration inhomogeneity factor below 2 m in height. In this paper, the space below 2 m in height is defined as the workspace.

**Figure 10:**
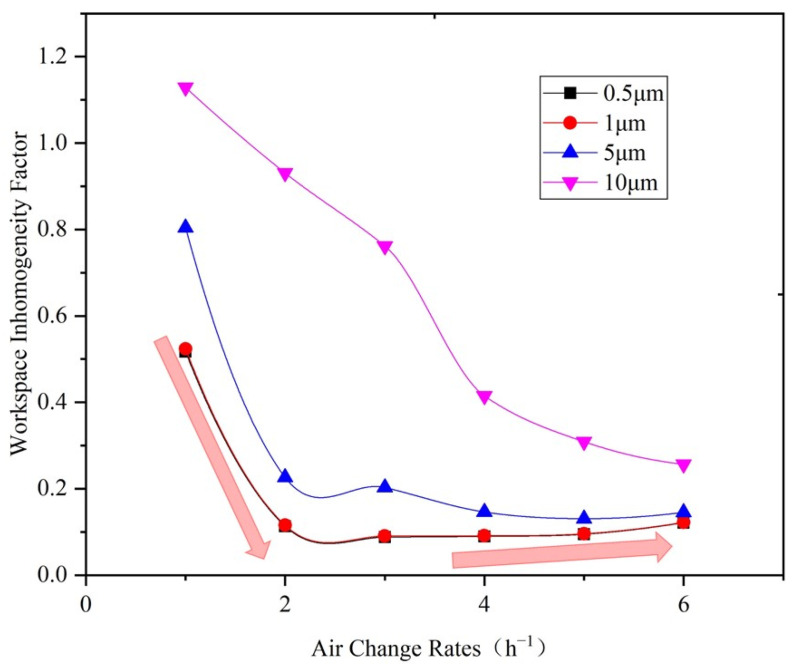
Relationship between the workspace inhomogeneity factor and air change rate.

Figure 10 shows the relationship between the workspace particle concentration inhomogeneity factor and the air change rate of the space under various particle size. It can be seen that the workspace inhomogeneity factor of 10 μm particles is 1.13 under air change rate (ACR) of one. With an increase in supply air volume, the workspace inhomogeneity factor declines rapidly, dropping to 0.26 when the ACR increases to 6. The workspace inhomogeneity factor of 5 μm particles decreases from 0.8 under 1 ACR to 0.13 under 5 ACR. Then, it increases slightly along with the air change rate. The variation trend of the workspace inhomogeneity factor for 0.5 μm and 1 μm particles is similar to that of 5 μm particles. The workspace inhomogeneity factor for 0.5 μm and 1 μm particles stops declining when the air change rate (ACR) is 3, and with a minimum of 0.09.

### 3.3. Distribution Indices of Particle Concentration

**Figure 11:**
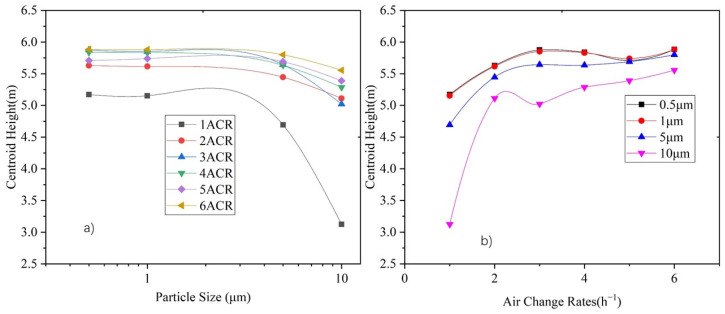
Variation of vertical distribution centroid height under 1–6 ACR for each particle size: (**a**) Relationship between centroid height and particle size, and (**b**) relationship between centroid height and air change rate.

The vertical particle concentration distribution centroid, diffusion radius, and stratification height, calculated by Equations (10)–(12), respectively, can be used to indicate the vertical distribution characteristics of particles. Figure 11 shows the vertical particle concentration distribution centroid of each particle size under various air supply volumes.

As Figure 11 shows, the distribution centroid is between 5 m and 6 m in height under an ACR of 2–6, and declines with an increase in the particle size. The supply air volume could increase the distribution centroid, especially for larger particles (i.e., 5 μm and 10 μm). The distribution centroid of 0.5 μm and 1.0 μm particles rises from 5.1 m to 5.8 m by supply air under an ACR from 1 to 6, respectively, whereas the centroid height of 5 μm and 10 μm particles rises from 4.7 m to 5.7 m and 3.1 m to 5.5 m, respectively.

**Figure 12:**
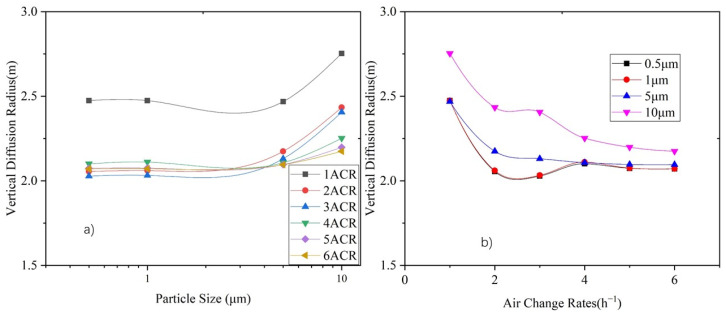
Variation in vertical diffusion radius height under ACR of 1–6 for each particle size. (**a**) Relationship between diffusion radius and particle size, and (**b**) relationship between diffusion radius and air changes rate.

Figure 12a shows the correlation between the vertical diffusion radius and particle size, and Figure 12b shows the correlation between the vertical diffusion radius and the ACR. It can be seen that the diffusion radius does not significantly change under different particle sizes, except for 10 μm particles. In terms of supply air volume, the diffusion radii of 0.5 μm, 1 μm, and 5 μm particles become stable (at around 2.1 m) under an ACR between 2 and 6, whereas the diffusion radius of 10 μm particles continuously decreases with an increase in the supply air volume.

**Figure 13:**
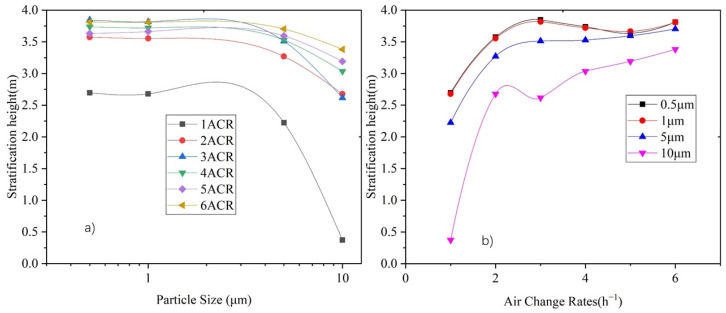
Variation of stratification height under 1–6 ACR for each particle size. (**a**) Relationship between stratification height and particle size, and (**b**) relationship between stratification height and air change rate.

The stratification height is obtained by subtracting the diffusion radius from the centroid height. Figure 13 shows the variation in the stratification height with respect to the particle size and air supply volume. It can be seen that when the ACR increases to 2, the particle distribution achieves stable stratification with stratification height is between 3 m and 4 m. The stratification height for particles smaller than 1 μm is basically the same, whereas it decreases with an increase in particle size. The stratification height for 10 μm is 0.2–0.8 m lower than that of particles smaller than 1 μm. 

The stratification height under various particle sizes increases with an increase in air supply volume. When the supply air volume reaches an air change rate of 2, the stratification height of particles smaller than 5 μm is not significantly influenced by the supply air volume, while the stratification height of 10 μm particles increases along with the air supply volume.

**Figure 14:**
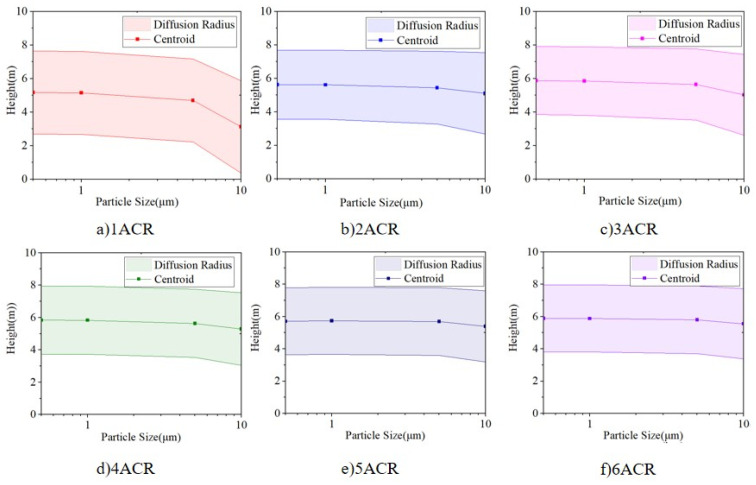
Relationship between centroid height, diffusion radius and stratification height at each air change rate with respect to the particle size: (**a**) ACR = 1, (**b**) ACR = 2, (**c**) ACR = 3, (**d**) ACR = 4, (**e**) ACR = 5, and (**f**) ACR = 6.

**Figure 15:**
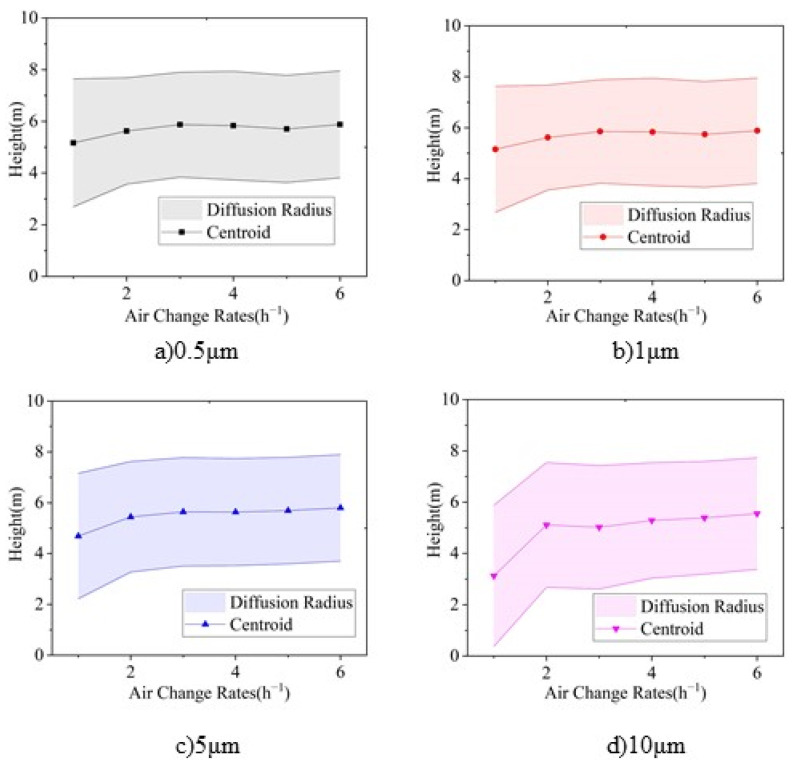
Relationship between centroid height, diffusion radius, and stratification height at each particle size with respect to the air change rate: (**a**) 0.5 μm, (**b**) 1 μm, (**c**) 5 μm, and (**d**) 10 μm.

For convenience, the variations in centroid height, diffusion radius, and stratification height for each air change rate along with the variation in particle size are combined into Figure 14, and that for each particle size along with the variation in air change rate are combined into Figure 15.

### 3.4. Horizontal Plane Diffusion Radius

**Figure 16:**
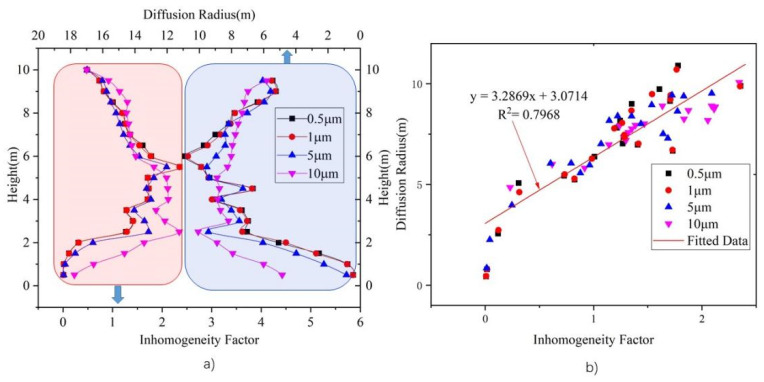
Relationship between vertical average inhomogeneity factor and horizontal plane diffusion radius. (**a**) Comparison of inhomogeneity factor with plane diffusion radius, and (**b**) correlation between inhomogeneity factor and horizontal plane diffusion radius.

The horizontal plane diffusion radius for each particle source was obtained by using Equation (13). Figure 16a shows a comparison of the inhomogeneity factor and horizontal plane diffusion radius under an ACR of 2. The curves in the red frame correspond to the left and bottom coordinate axis, whereas those in the blue frame correspond to the right and top coordinate axis. It can be seen that planes with a high inhomogeneity factor also have a large horizontal diffusion plane radius. Figure 16b shows the correlation between the inhomogeneity factor and horizontal plane diffusion radius. The coordinates of the points are the inhomogeneity factor and plane diffusion radius, respectively, and the red line shows the fitted line. A direct correlation can be observed, with R^2^ = 0.7968. Thus, it can be inferred that a large plane diffusion radius means that particles reach and accumulate at that level, leading to a high inhomogeneity factor.

### 3.5. Sensitivity Analysis of Particle Concentration Distribution

**Figure 17:**
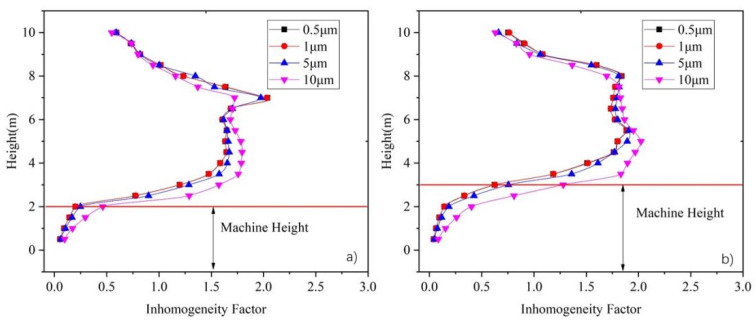
Inhomogeneity factor distribution for difference machine heights under ACR = 6. (**a**) Machine height of 2 m, and (**b**) machine height of 3 m.

The workspace inhomogeneity factor and vertical particle concentration stratification height are fundamental indices for the design and control of ventilation systems in engineering applications. Many parameters influence these two indices, including the supply air volume, particle size, machine surface temperature, machine height, and supply air temperature. Figure 17 shows the influence of machine height on the vertical inhomogeneity factor under an ACR of 6. Therefore, it is necessary to analyze the sensitivity of these two indices to these parameters. The orthogonal experimental method [61] was employed for this purpose.

The involved parameters were supply air volume, supply air temperature, surface temperature of production unit, and height of production unit. Three levels were set for each parameter. The orthogonal experimental code table, consisting of 4 factors and 3 levels, is shown in Table 3, whereas the stratification height and workspace inhomogeneity factor for each code are shown in Table 4 and Table 5, respectively.

The range analysis method [62] was used to conduct the sensitivity analyses for the stratification height and workspace inhomogeneity factor at various particle sizes. The analysis process for the stratification height of 0.5 μm particles is shown in Table 6.

*K*1, *K*2, and *K*3 in the table are the algebraic sums of the experimental results at level 1, level 2, and level 3 of each parameter, respectively; for example, *K*2 with respect to the supply air temperature is equal to the sum of stratification heights under a supply air temperature of 26 °C. $\bar{K1}$, $\bar{K2}$, and $\bar{K3}$ in the table are the average values of the experimental results at level 1, level 2, and level 3 of each parameter; for example, $\bar{K2}$ is equal to *K2* divided by the number of levels. The range R in the table is the difference between the maximum and minimum values in $\bar{K1}$, $\bar{K2}$, and $\bar{K3}$. The larger the range, the higher the sensitivity of the experimental results to the experimental parameters.

From Table 6, it can be seen that the stratification height for 0.5 μm particles is sensitive to the parameters, in descending order, as follows: machine height, machine surface temperature, air supply volume, and air supply temperature.

The workspace inhomogeneity factor for 0.5 μm particles is most sensitive to the machine height, followed by the supply air volume, machine surface temperature, and supply air temperature, respectively. For the sake of space, the other analysis processes are not given here; however, details are provided in the Supplementary Materials (see Tables S4–S10).

The sensitivity analysis summaries for the workspace inhomogeneity and the stratification height at each particle size are given in Table 7, from which it can be seen that the stratification height under small particle sizes (<5 μm) is more sensitive to machine height and machine surface temperature than the other parameters, whereas the stratification height under large particle sizes (≥5 μm) is more sensitive to the supply air volume than the other parameter. Furthermore, the workspace inhomogeneity factor under small particle sizes (<5 μm) is more sensitive to machine height, whereas that under large particle sizes (≥5 μm) is more sensitive to the supply air volume.

## 4. Discussion

### 4.1. Vertical Distribution of Particle Concentration Inhomogeneity

**Figure 18:**
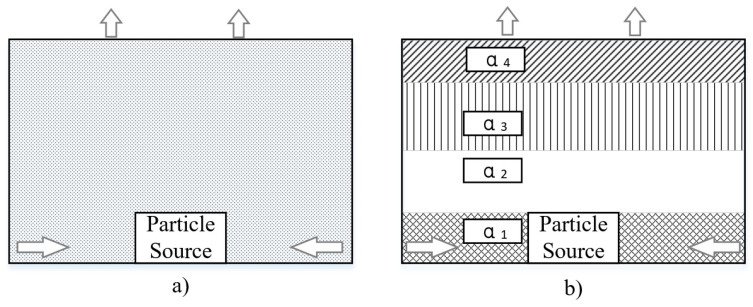
Principle of homogeneous and inhomogeneous particle concentration distribution. (**a**) Homogeneous distribution, and (**b**) inhomogeneous distribution.

The vertical distribution of the particle concentration inhomogeneity factor can indicate the relative distribution of particles generated from indoor particle sources under the action of a ventilation system. Figure 18 depicts the principle of homogeneous and inhomogeneous distributions.

The indoor particle concentration, supply air volume and particle source emission rate are governed by Equation (14), without considering an inhomogeneous particle concentration distribution:

(14) $$\begin{array}{c} C_{i\_indoor}=\frac{E_{i}+Q_{sa}\times C_{i\_s}}{Q_{sa}}=\frac{E_{i}}{Q_{sa}}+C_{i\_s}, \end{array}$$

where $C_{i\_indoor}$ is the indoor average concentration of particles of size *i* (mg·m^−3^), $C_{i\_s}$ is the concentration of particles of size *i* in the supply air (mg·m^−3^), $E_{i}$ is the emission rate of particles of size *i* from indoor sources (mg·h^−1^), and $Q_{sa}$ is the supply air volume (m^3^·h^−1^).

Then, the supply air volume to control the indoor particle concentration can be calculated as

(15) $$\begin{array}{c} Q_{sa}=\frac{E_{i}}{C_{i\_indoor}-C_{i\_s}}. \end{array}$$

If an inhomogeneous distribution cannot be ignored—for example, when considering the particle concentration in a large-space workshop under displacement ventilation—the particle concentration should be calculated by using

(16) $$\begin{array}{c} C_{i_{\_}indoor\_k}=\frac{\alpha_{i\_k}\times E_{i}+Q_{sa}\times C_{i\_s}}{Q_{sa}}=\alpha_{i\_k}\times \frac{E_{i}}{Q_{sa}}+C_{i\_s}, \end{array}$$

where $C_{i\_indoor\_k}$ is the average concentration in block *k* of particles of size *i* (mg·m^−3^), and $\alpha_{i\_k}$ is the particle concentration inhomogeneity factor of block *k* under particles of size *i*.

Then, the supply air volume to control the particle concentration in block *k* can be calculated as

(17) $$\begin{array}{c} Q_{sa}=\frac{\alpha_{i\_k}\times E_{i}}{C_{i\_indoor\_k}-C_{i\_s}}. \end{array}$$

Comparing Equation (15) with Equation (17), it can be inferred that if inhomogeneity cannot be ignored, the supply air volume required to control particle concentration is correlated with $\alpha_{i\_k}$. When $\alpha_{i\_k}$ is less than 1, the supply air volume necessary to control the particle concentration in a specified block is less than that in the situation where an inhomogeneous distribution is not considered; however, if $\alpha_{i\_k}$ is greater than 1, the situation is the opposite. The inhomogeneity factor is, therefore, a good index with which to correct the supply air volume calculation when partial spatial particle concentration control is needed. With the emission rate of the indoor particle source and inhomogeneity factor distribution rules, the ventilation volume of DV, which was used to control the particle concentration of specified block, can be calculated by using Equation (17).

Generally, the average vertical inhomogeneity factor in the lower part of the considered large-space workshop was found to be less than 1, whereas that in the middle part was greater than 1 under the DV system. This means that the lower part of the large-space workshop is cleaner than the middle part under the DV system. Therefore, the necessary supply air volume to control the particle concentration in the lower part in the large-space workshop under a DV system is less than that required when using a mixing ventilation system. Zhang at el. [34], Wei at el. [35], and Wang at el. [37] have all reached the similar conclusions.

According to Figure 10, the workspace inhomogeneity factor for particles smaller than 5 μm is less than 0.25 under the DV system when the ACR is in the range of 2–6. Thus, through conservative calculations, controlling the workspace particle concentration under the DV system needs no more than 1/3 of the supply air volume of a mixing ventilation system, if there are only small particles (≤5 μm). However, for large particles (e.g., 10 μm particles), the workspace particle concentration inhomogeneity factor is much greater than that of small particles, due to the effect of gravity. Therefore, the required supply air volume to control large particle concentrations is almost twice that of small particles. However, oil particles in machining workshops typically have a wide size range. It is necessary to choose the inhomogeneity factor corresponding to the particle size. If larger particles can be captured by local equipment, the demanded supply air volume for the DV system will be significantly reduced. Such a combined ventilation system needs to be further investigated.

Wang et al. [59,60] have developed an oil particle emission rate prediction model for a milling process. If there are plenty of models available to predict particle source emission rate, the particle concentration inhomogeneity factor can be used to control the operation of the DV system.

### 4.2. Distribution Indices of Particle Concentration

The vertical particle concentration distribution centroid, diffusion radius, and stratification height, which are based on the first- and second-order moments of the vertical concentration distribution, are good indicators reflecting the vertical particle concentration under a ventilation system. As such, these indices can be applied for the optimization of ventilation systems or airflow patterns.

The horizontal plane diffusion radius has a direct correlation with the inhomogeneity factor. A high plane diffusion radius reveal that particles carried by the heat plume stop rising and begin to spread in a horizontal direction. The plane diffusion radius can also be used as a ventilation optimization indicator, where a low plane diffusion radius indicates good particle source control ability.

### 4.3. Sensitivity Analysis of Particle Concentration Distribution

According to the results of the sensitivity analysis, the stratification height of particles smaller than 5 μm was most sensitive to the machine height, whereas that of particles larger than 5 μm was more sensitive to the supply air volume. The possible reason for this is that small (<5 μm) particles are less affected by gravity and, so, have lower particle settling velocity. The particle source was considered to be on the top of the machines. Once small (<5 μm) particles are emitted from the machine, they will be carried by the heat plume generated by the machines. However, the average velocity of the heat plume is not high enough to carry large particles (≥5 μm), such that the supply air volume has a greater effect on increasing the stratification height of these particles. The sensitivity of the workspace inhomogeneity factor follows the same rules, possibly for the same reasons.

It can be inferred that, if small particles are the major contamination, a baffle—which can increase the machine height—is a good solution for raising the stratification height and reducing the workspace inhomogeneity factor. Moreover, if large particles are the major contamination, increasing the supply air volume is a more effective method.

Although, the supply air temperature was not the most sensitive parameter for the stratification height and workspace inhomogeneity factor, it still can influence these two indices. A possible reason for this is that the supply air temperature influences the heat plume of the machine indirectly, which influences the particle distribution, stratification height, and workspace inhomogeneity factor.

Many other factors may influence the inhomogeneity factor and stratification height which may not be major influencing factors; however, a more detailed computational model is necessary to obtain an accuracy vertical inhomogeneity factor.

### 4.4. Material of Oil Particles

Few papers have studied the chemical composition of airborne particle in machining workshops directly. Zhang et al. [22] have studied the physicochemical characterization of oily particles emitted from different machining processes in an industrial plant, and showed that over 80% of the content of the particles is oil. Although the chemical composition was not studied, they reported that oil particles were emitted from MWFs in the machining process. Thus, the major chemical component of oil particles should be similar to that of MWFs.

There are basically two types of MWFs: oil-based MWFs and water-soluble MWFs [63]. Oil-based MWFs are a mixture of mineral oil (major component, consisting of various alkane such as hexadecane, octadecane, and tetradecane) and other components, including chlorinated paraffins, compounds containing sulphur, tricresylphosphates, and so on. Water-soluble MWFs consist of petroleum or mineral oil in combination with emulsifying agents and additives, acting in the form of an emulsion which is diluted by water.

Cooper et al. [64] have studied the evaporation of MWFs mist in industrial mist collectors. Their results showed that 10% of the mineral oils and 1.4% of the soluble oil emulsion would change to vapor phase under standard conditions in 5 days—equivalent to a reduction in particle size less than 5%. In this study, the minimum ventilation volume changed the workshop indoor space air at least one time per hour. Thus, the size reduction due to the evaporation of oil particles can be ignored in the period in which the oil particles are suspended in the indoor environment. 

As for water evaporation of water-soluble MWFs, Wang et al. [60] have found that different dilution ratios do not lead to a noticeable change in emission particle size distribution, and concluded that water evaporation in the MWFs droplets is completed in the inner part of the machine. Thus, from the moment oil particle leaves the machine units, its size will not be reduced further.

Therefore, size reduction due to the evaporation of oil was ignored in this study, and the oil particles were set as inert particles in the CFD simulations.

We focused on oil particles, which have several specific characteristics: (1) Oil particles are spherical due to surface tension and, so, the drag force computation is different than that for solid particulate matter; (2) oil particles have a density much lower than that of solid particulate matter, so the gravity and deposition of oil particles is quite different than solid particulate matter; and (3) once oil particles reach a solid surface, they tend to stick to the surface, whereas some solid particulate matter may rebound in various directions. Therefore, the results and conclusions obtained herein cannot be generalized to solid particulate matter.

## 5. Conclusions

In this study, we proposed an inhomogeneity factor which can describe the relative oil particle concentration distribution for various particle sizes under the use of a DV system. Through CFD simulation, we found that the particle concentration inhomogeneity factor distribution shows similar rules along the vertical direction under a DV system with an ACR of 2–6, and the particle concentration below 3 m is significantly lower than that at middle height (i.e., between 3 m and 8 m in height).

With the inhomogeneity factor distribution data and emission data for particle sources, the supply air volume of the DV system can be corrected, according to the height at which the particle concentration needs to be controlled.

The workspace inhomogeneity factor of particles smaller than 5 μm is less than 0.25, such that controlling the workspace particle concentration with a DV system requires no more than 1/3 that of the supply air volume of a mixing ventilation system if only small particles (<5 μm) are present. Around double the supply air volume is needed to maintain the 10 μm particle concentration at same level as that of particles smaller than 5 μm under the DV system.

The workspace inhomogeneity factor of small particles (<5 μm) is more sensitive to the machine height and machine surface temperature than other parameters, whereas that of large particles (>5 μm) is more sensitive to the supply air volume. Therefore, raising the machine height provides a good solution for reducing the workspace inhomogeneity factor of small particles (<5 μm); for large particles, increasing the supply air volume is more effective.

## Figures and Tables

**Table 1:** CFD model selection, settings and inputs.

Type	Model Selection	Settings and Inputs	Reference
Models	Energy	Energy Equation On	
	Viscous	RNG k-ε (two equations) Standard Wall Function Fluent Default Constants	Zhang et al. [34] Wei et al. [35]
	Discrete Phase	Method: DPM Particle Type: Inert Material: Fuel–Oil–Liquid Physical Models: Spherical Turbulent Dispersion: DRW Number of Tries: 100 Force: Drag force, Gravity Interaction: No Wall Boundary Type: Trap	Zhang et al. [34] Wei et al. [35] Zhang et al. [45]
Material	Air	Density: Boussinesq hypothesis Other Properties: Default Constants	Chen et al. [47] Liu et al. [48] Liu et al. [49] Zhao et al. [50]
Solution Methods	Pressure-Velocity Coupling	SIMPLE	Zhao et al. [39] Zhang et al. [46]
	Pressure:	PRESTO!	
	Momentum:	Second-order upwind method	
	Turbulent Kinetic Energy:	Second-order upwind method	
	Turbulent Dissipation Rate:	Second-order upwind method	
	Energy:	Second-order upwind method	

**Table 2:** CFD boundary condition information.

Type	Location	Parameter	Type of Boundary Condition
Surface Boundary Condition	Roof	39 °C	Dirichlet
	Wall	33 °C	Dirichlet
	Machine	35 °C	Dirichlet
	Ground	0 W·m^−2^	Neumann
Supply Air Vents	Velocity Inlet	0.083–0.5 m·s^−1^	Turbulence Intensity 10%
	Temperature	22 °C	Velocity Inlet
Air Outlet	Velocity Outlets	Corresponding to Supply Air	Velocity Outlets Turbulence Intensity 10%
Particle Source	Emission Rate	1 × 10^−6^ kg·s^−1^	Uniform at Machine Surface
	Particle Size	0.5 μm, 1.0 μm, 5 μm, and 10 μm	

**Table 3:** Orthogonal experimental code information.

Number	Supply Air Velocity (m·s^−1^)/ Air Change Rate (ACR)	Air Supply Temperature (°C)	Machine Height (M)	Machine Surface Temperature (°C)	Orthogonal Code
1	0.083/1	22	1.5	32	A_1_B_1_C_1_D_1_
2	0.083/1	26	2.0	35	A_1_B_2_C_2_D_2_
3	0.083/1	30	3.0	37	A_1_B_3_C_3_D_3_
4	0.250/3	22	2.0	37	A_2_B_1_C_2_D_3_
5	0.250/3	26	3.0	32	A_2_B_2_C_3_D_1_
6	0.250/3	30	1.5	35	A_2_B_3_C_1_D_2_
7	0.500/6	22	3.0	35	A_3_B_1_C_3_D_2_
8	0.500/6	26	1.5	37	A_3_B_2_C_1_D_3_
9	0.500/6	30	2.0	32	A_3_B_3_C_2_D_1_

Note: A stands for air supply volume, B stands for air supply temperature, C stands for machine tool height, and D stands for machine surface temperature.

**Table 4:** Stratification height for each particle size at various orthogonal experimental codes.

Orthogonal Code	Stratification Height of 0.5 μm (m)	Stratification Height of 1 μm (m)	Stratification Height of 5 μm (m)	Stratification Height of 10 μm (m)
A_1_B_1_C_1_D_1_	2.27	2.25	1.66	0.88
A_1_B_2_C_2_D_2_	2.89	2.86	2.42	1.42
A_1_B_3_C_3_D_3_	4.23	4.22	3.89	3.12
A_2_B_1_C_2_D_3_	3.94	3.93	3.76	3.02
A_2_B_2_C_3_D_1_	3.84	3.83	3.55	2.90
A_2_B_3_C_1_D_2_	3.55	3.56	3.41	2.86
A_3_B_1_C_3_D_2_	4.28	4.28	4.13	3.71
A_3_B_2_C_1_D_3_	3.61	3.60	3.47	3.15
A_3_B_3_C_2_D_1_	3.55	3.54	3.47	3.25

Note: A stands for air supply volume, B stands for air supply temperature, C stands for machine tool height, and D stands for machine tool surface temperature.

**Table 5:** Workspace inhomogeneity factor for each particle size at various orthogonal experimental codes.

Orthogonal Code	0.5 μm Inhomogeneity Factor of Workspace	1 μm Inhomogeneity Factor of Workspace	5 μm Inhomogeneity Factor of Workspace	10 μm Inhomogeneity Factor of Workspace
A_1_B_1_C_1_D_1_	0.70	0.70	0.85	0.77
A_1_B_2_C_2_D_2_	0.50	0.50	0.65	0.81
A_1_B_3_C_3_D_3_	0.09	0.09	0.20	0.59
A_2_B_1_C_2_D_3_	0.07	0.07	0.14	0.64
A_2_B_2_C_3_D_1_	0.12	0.13	0.22	0.64
A_2_B_3_C_1_D_2_	0.19	0.19	0.29	0.69
A_3_B_1_C_3_D_2_	0.08	0.09	0.11	0.22
A_3_B_2_C_1_D_3_	0.30	0.31	0.35	0.51
A_3_B_3_C_2_D_1_	0.38	0.38	0.41	0.51

**Table 6:** Process of 0.5 μm stratification height range analysis in the orthogonal experiment.

Orthogonal Code	Supply Air Velocity (m·s^−1^)/ Air Change Rate (ACR)	Supply Air Temperature (°C)	Machine Height (m)	Machine Surface Temperature (°C)	0.5 μm Stratification Height (m)
A_1_B_1_C_1_D_1_	0.083/1	22	1.5	32	2.27
A_1_B_2_C_2_D_2_	0.083/1	26	2.0	35	2.89
A_1_B_3_C_3_D_3_	0.083/1	30	3.0	37	4.23
A_2_B_1_C_2_D_3_	0.250/3	22	2.0	37	3.94
A_2_B_2_C_3_D_1_	0.250/3	26	3.0	32	3.84
A_2_B_3_C_1_D_2_	0.250/3	30	1.5	35	3.55
A_3_B_1_C_3_D_2_	0.500/6	22	3.0	35	4.28
A_3_B_2_C_1_D_3_	0.500/6	26	1.5	37	3.61
A_3_B_3_C_2_D_1_	0.500/6	30	2.0	32	3.55
*K*1	9.38	10.49	9.42	9.65	-
*K*2	11.33	10.33	10.38	10.72	-
*K*3	11.44	11.32	12.34	11.77	-
$\bar{K1}$	3.13	3.50	3.14	3.22	-
$\bar{K2}$	3.78	3.44	3.46	3.57	-
$\bar{K3}$	3.81	3.77	4.11	3.92	-
Range	0.69	0.33	0.97	0.71	-
Sort	C > D > A > B				

Note: A stands for air supply volume, B stands for air supply temperature, C stands for machine tool height, and D stands for machine tool surface temperature.

**Table 7:** Sensitivity sorting information of influence factors of stratification height and workspace inhomogeneity factor.

Particle Size (μm)	Sensitivity Ranking for Stratification Height	Sensitivity Ranking for Workspace Inhomogeneity Factor
0.5	C > D > A > B	C > A > D > B
1.0	C > D > A > B	C > A > D > B
5.0	A > C > D > B	A > C > D > B
10.0	A > C > D > B	A > C > D > B

Note: A stands for air supply volume, B stands for air supply temperature, C stands for machine tool height, and D stands for machine tool surface temperature.

## Data Availability

The data presented in this study are available in the article and Supplementary Materials.

## Supplementary Materials

The following are available online at https://www.mdpi.com/article/10.3390/ijerph19116932/s1, Figure S1: Structural diagram of oil mist particle emission device. Figure S2: Schematic diagram of emission rate test device. Table S1: Comparison of metal working fluids and aerosol solvent parameters. Table S2: Result of aerosol generator emission rate test. Table S3: Boundary conditions used in CFD validation experiment. Table S4: Orthogonal analysis table of workspace inhomogeneity factor for 0.5 μm particles. Table S5: Orthogonal analysis table of stratification height for particles of size 1 μm. Table S6: Orthogonal analysis table of workspace inhomogeneity factor for particles of size 1 μm. Table S7: Orthogonal analysis table of stratification height for particles of size 5 μm. Table S8: Orthogonal analysis table of workspace inhomogeneity factor for particles of size 5 μm. Table S9: Orthogonal analysis table of stratification height for particles of size 10 μm. Table S10: Orthogonal analysis table of workspace inhomogeneity factor for particles of size 10 μm.
