# Supplementary material for: Study of Oil Particle Concentration Vertical Distribution of Various Sizes under Displacement Ventilation System in Large-Space Machining Workshop
Source: Int J Environ Res Public Health. 2022 Jun 6;19(11):6932. doi: 10.3390/ijerph19116932 (PMC9180901; doi:10.3390/ijerph19116932)
Supplement: Supplementary file 1 [file ijerph-19-06932-s001.zip › ijerph-1715904-supplementary.pdf]

International Journal of
Environmental Research and Public Health

MDPI

Supplementary Information

S1. Details of CFD Validation

S1.1 Details of Experimental System

The air supply system of the air conditioner consists of three parts: A nozzle side air supply system, a column-down air supply system, and an airbag supply system. In this experiment, the column-down air supply is selected. There are eight column-down air supply inlets, which are divided into two groups and installed in the south wall and the north wall, respectively. The column-down air supply inlets are 1 m in diameter and 1.5m in height.

S1.2. Experimental Site and Conditions

We measured the temperature and particle concentration data of line 1, line 2, and line 3. The CFD simulation data for line 1, line 2, and line 3 are compared for validation. Fig. S2 shows the position of the test line and emission source.

In the experimental process, the supply air volume of displacement ventilation (DV) system was 23,500$m^3 \cdot h^{-1}$, which was measured using a test tube in the supply air duct. Each columnar air supply inlet was adjusted to the same supply air volume by dampers. The exhaust air outlet was set in the lower part of the east wall, with a specific size of 2.9m (width) and 2.0m (height). The bottom edge of the exhaust air outlet was 0.5m from the ground. Two variable frequency exhaust fans were set on top of the building. Due to the limitations of the exhaust fan ability, the exhaust air volume from the top was 4700 $m^3 \cdot h^{-1}$, and the exhaust air from the outlet near the ground was 18,800 $m^3 \cdot h^{-1}$.

In this study, a particle counter (Lighthouse IAQ 3016) was used to measure particle concentration on each test line in the validation experiment, and the emission rate test of the particle source. The particle counter contain 6 particle size channels: 0.3μm, 0.5μm, 1.0μm, 2.5μm, 5.0μm, 10.0μm, and concentration accuracy of particles under size greater than 0.5μm is ±10%, while that of 0.3μm particles is ±20%.

S1.3. Oil Particle Source and Emission Rate Test Method

As the machine cannot run for a long time to generate oil particles, we employed an aerosol generator instead, which can be seen in Fig. S1. The aerosol-generating device consists of an aerosol generator and an aerosol current equalizing device. The model of the aerosol generator is an ATI(TDA-4B).

An aerosol flow-equalizing device was employed to make the aerosol characteristics and initial state close to real situation. The particle emission height was 1.5m, close to the position of the machine emission point.

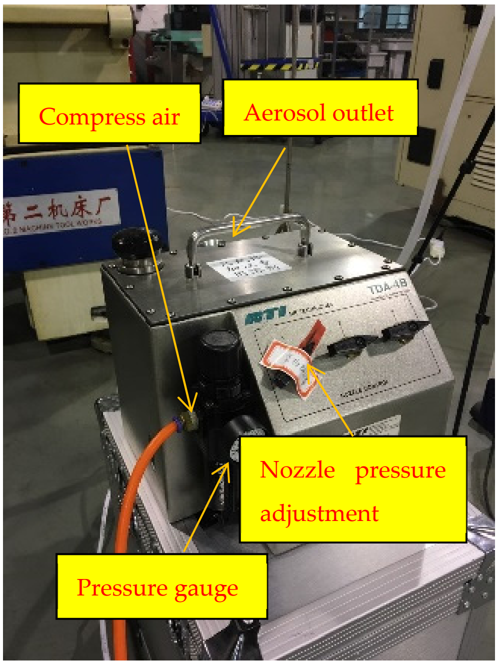

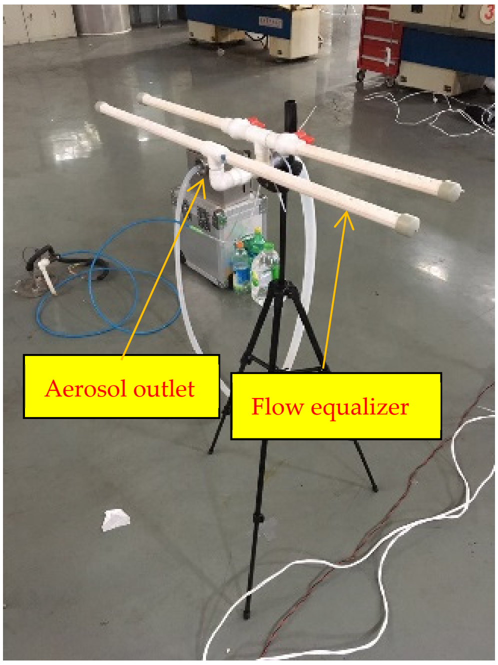

(a)Aerosol generator (b)Aerosol generating and equalizing device

Figure S1. Structural diagram of oil mist particle emission device.

In order to make the particles in this experiment consistent with those emitted by machines, and to ensure the health and safety of laboratory person, an ATI-PAO aerosol solvent (polyolefin oil Ploy Olefin) was used in this experiment. Table S1 shows the comparison between the properties of the solvents used in the experiment and those of fluids commonly used in the machining process.

Table S1. Comparison of metal working fluids and aerosol solvent parameters

Properties	Mobil 2 Cutting Oil	ATI-PAO Testing oil
Density	0.889	0.819
Kinematic Viscosity ($mm^2 \cdot s^{-1}$)	31.5	30.4
Dynamic viscosity (mPa·s)	28.0	24.9
Viscosity index (VI)	118	117
Pour point (℃)	−33	−45
Flash point (℃)	228	242
Water-solubility	Insoluble	Insoluble

The emission rate of the aerosol generator was tested using the device shown in Fig. S2. The outlet of the fan was connected with the air volume test chamber through an air duct. The air volume test chamber contains a combined nozzle, and its structure is in accordance with the type C air chamber in ISO 5801:2007 [51]. Particles generated by the aerosol generator were injected into the outlet duct of the wind chamber. A particle detector was set in the duct, where the oil particles were completely diffused. A high-efficiency

filter was installed after the particle detector to filter the particles in the duct.

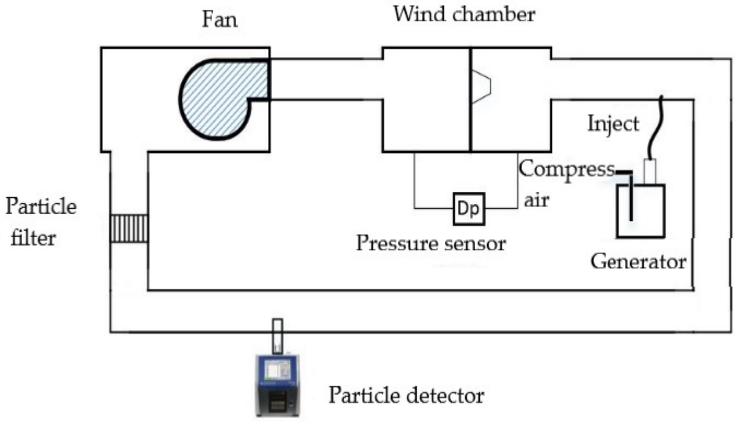

Figure S2. Schematic diagram of emission rate test device

The emission rate of oil particles was calculated by equation (S1):

$$\mathrm{E}_i = \mathrm{Q}_S C_i, \quad \text{(S1)}$$

where E_i is the emission rate of oil particles of size *i* (μg·s^{-1}); Q_S is the total flow of the nozzle system (m^3·s^{-1}), which can be obtained from differential pressure of the nozzle according to ISO 5801:2007 [51]; and C_i is the aerosol concentration of the test point particles of size *i* (μg·m^3).

The result of the aerosol generator emission rate test is shown in Table S2, and the particle size distribution of particles from the aerosol generator is shown in Fig. S3. The results were similar to the particles emitted due to machine processing [59]. The aerosol generator was adjusted to the same pressure and nozzle position as in the experiment with the emission rate test.

Table S2. Result of aerosol generator emission rate test

**Grain size (μm)**	0.3	0.5	1.0	2.5	5.0	10	Total Particulate Mass
**Dust emission (μg/s)**	3.84	33.75	409.69	896.26	99.51	0.27	1443.32
**Standard deviation (μg/s)**	0.02	0.06	1.56	9.21	2.31	0.09	10.40
**Proportion (%)**	0.02	2.34	28.39	62.10	6.90	0.02	100.00

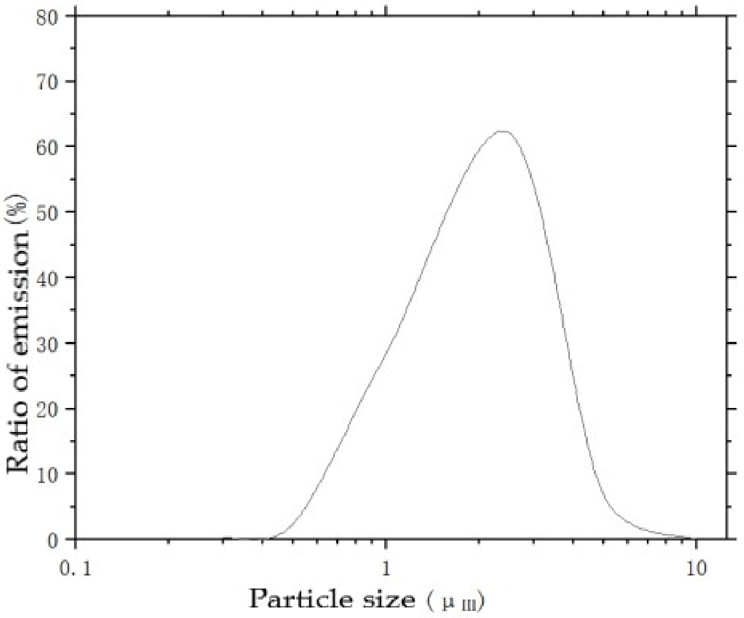

Figure S3. Particle size distribution of particles from aerosol generator

S1.4. Experimental Steps and Data Processing

The experiment was carried out as follows:

1. Switch on the supply fan with running frequency at 50Hz. At this time, the measured air volume of the system is 23,500 $m^3 \cdot h^{-1}$. Then, adjust the exhaust fan on the roof and exhaust outlet to keep supply air and exhaust air balanced.

2. After the indoor temperature is stabilized, test the particle concentration at each measurement point.

3. Switch on the aerosol generator and adjust the oil mist emission pressure and nozzle flow to keep it at the same state as the emission rate test. Begin monitoring the particle concentration of the exhaust air outlet.

4 When the particle concentration in the return air outlet is stable, measure the oil particle concentration and temperature at each measurement point. The temperature measurement point heights are in the range of 0.2-11 m, and 11 measuring points are evenly arranged on each test line. The particle concentration test points are at 1.5 m, 3.0 m, and 7 m height on the test line, respectively.

The indoor particle concentration was generalized using equation (S2)

$$C_{i\_g} = \frac{C_{i\_indoor} - C_{i\_b}}{C_{i\_tm}}, \tag{S2}$$

where C_{i_g} is the (dimensionless) concentration of particles of size i; C_{i_indoor} is the measured concentration of particles of size i ($mg \cdot m^{-3}$); C_{i_b} is the background concentration of particles of size i ($mg \cdot m^{-3}$), which was determined in step 2; C_{i_tm} is the instantaneous homogeneous diffused concentration of particles of size i ($mg \cdot m^{-3}$), which can be obtained by equation S3

$$C_{i\_tm} = \frac{E_i}{Q_{sa}}, \quad \text{(S3)}$$

where E_i is the particle emission rate of particles of size i (mg·s^{-1}) and Q_{sa} is the air supply volume of the system (m^3·s^{-1}).

S1.5. Boundary Conditions of CFD for Validation Case

The boundary conditions used for CFD were set according to the measured data. Detailed information can be found in Table S3. In this study, 1 μm particles were chosen as validation particles and the emission rate in CFD was consistent with the test emission rate.

Table S3. Boundary conditions used in CFD validation experiment

Type	Location	Parameter	Type of boundary condition
Surface Boundary Condition	East wall	28.3 ℃	Dirichlet
	South wall	30.5 ℃	Dirichlet
	West wall	30.6 ℃	Dirichlet
	North wall	30.6 ℃	Dirichlet
	Roof	40.0 ℃	Dirichlet
	Ground	0 W·m^{-2}	Neumann
	Machine tool	35 ℃	Dirichlet
Supply Air	Inlets	0.346 m·s^{-1}	Turbulence Intensity 10%
	Temperature	26 ℃	Velocity Inlets
Exhaust Air	Outlets Near Ground	0.866 m·s^{-1}	Turbulence Intensity 10% Velocity Outlets
Exhaust Air	Roof	4700 m^3·h^{-1}	Turbulence Intensity 10% Velocity Outlets
Particle Source	Emission Rate	0.41 × 10^{-6}kg·s^{-1}	Single Point Source
	Particle Size	1.0 μm	

S2. Sensitivity Analysis Data

Table S4. Orthogonal analysis table of workspace inhomogeneity factor for 0.5μm particles

Orthogonal Code	Supply Air Velocity ($m·s^{-1}$) /Air Change Rate (ACR)	Supply Air Tempera-ture (℃)	Machine Height (m)	Machine Surface Tempera-ture (℃)	0.5μm Inhomogeneity Factor of Workspace
$A_1B_1C_1D_1$	0.083/1	22	1.5	32	0.70
$A_1B_2C_2D_2$	0.083/1	26	2.0	35	0.50
$A_1B_3C_3D_3$	0.083/1	30	3.0	37	0.09
$A_2B_1C_2D_3$	0.250/3	22	2.0	37	0.07
$A_2B_2C_3D_1$	0.250/3	26	3.0	32	0.12
$A_2B_3C_1D_2$	0.250/3	30	1.5	35	0.19
$A_3B_1C_3D_2$	0.500/6	22	3.0	35	0.08
$A_3B_2C_1D_3$	0.500/6	26	1.5	37	0.30
$A_3B_3C_2D_1$	0.500/6	30	2.0	32	0.38
K1	1.29	0.85	1.19	1.21	-
K2	0.38	0.93	0.95	0.77	-
K3	0.77	0.66	0.30	0.46	-
$\overline{K1}$	0.43	0.28	0.40	0.40	-
$\overline{K2}$	0.13	0.31	0.32	0.26	-
$\overline{K3}$	0.26	0.22	0.10	0.15	-
Range	0.30	0.09	0.30	0.25	-
sort	C>A>D>B				

Table S5. Orthogonal analysis table of stratification height for particles of size 1μm

Orthogonal Code	Supply Air Velocity ($m\cdot s^{-1}$) /Air Change Rate (ACR)	Supply Air Temperature (℃)	Machine Height (m)	Machine Surface Temperature (℃)	1μm Stratification Height (m)
$A_1B_1C_1D_1$	0.083/1	22	1.5	32	2.25
$A_1B_2C_2D_2$	0.083/1	26	2.0	35	2.86
$A_1B_3C_3D_3$	0.083/1	30	3.0	37	4.22
$A_2B_1C_2D_3$	0.250/3	22	2.0	37	3.93
$A_2B_2C_3D_1$	0.250/3	26	3.0	32	3.83
$A_2B_3C_1D_2$	0.250/3	30	1.5	35	3.56
$A_3B_1C_3D_2$	0.500/6	22	3.0	35	4.28
$A_3B_2C_1D_3$	0.500/6	26	1.5	37	3.60
$A_3B_3C_2D_1$	0.500/6	30	2.0	32	3.54
K1	9.33	10.45	9.41	9.62	-
K2	11.32	10.29	10.34	10.70	-
K3	11.42	11.32	12.32	11.75	-
$\overline{K1}$	3.11	3.48	3.14	3.21	-
$\overline{K2}$	3.77	3.43	3.45	3.57	-
$\overline{K3}$	3.81	3.77	4.11	3.92	-
Range	0.70	0.34	0.97	0.71	-
sort	C>D>A>B				

Table S6. Orthogonal analysis table of workspace inhomogeneity factor for particles of size 1μm

Orthogonal Code	Supply Air Velocity ($m \cdot s^{-1}$) /Air Change Rate (ACR)	Supply Air Temperature (℃)	Machine Height (m)	Machine Surface Temperature (℃)	1μm Inhomogeneity Factor of Workspace
$A_1B_1C_1D_1$	0.083/1	22	1.5	32	0.70
$A_1B_2C_2D_2$	0.083/1	26	2.0	35	0.50
$A_1B_3C_3D_3$	0.083/1	30	3.0	37	0.09
$A_2B_1C_2D_3$	0.250/3	22	2.0	37	0.07
$A_2B_2C_3D_1$	0.250/3	26	3.0	32	0.13
$A_2B_3C_1D_2$	0.250/3	30	1.5	35	0.19
$A_3B_1C_3D_2$	0.500/6	22	3.0	35	0.09
$A_3B_2C_1D_3$	0.500/6	26	1.5	37	0.31
$A_3B_3C_2D_1$	0.500/6	30	2.0	32	0.38
K1	1.30	0.86	1.20	1.21	-
K2	0.39	0.93	0.95	0.78	-
K3	0.77	0.67	0.30	0.47	-
$\overline{K1}$	0.43	0.29	0.40	0.40	-
$\overline{K2}$	0.13	0.31	0.32	0.26	-
$\overline{K3}$	0.26	0.22	0.10	0.16	-
Range	0.30	0.09	0.30	0.25	-
sort	C>A>D>B				

Table S7. Orthogonal analysis table of stratification height for particles of size 5μm

Orthogonal Code	Supply Air Velocity ($m\cdot s^{-1}$) /Air Change Rate (ACR)	Supply Air Temperature (℃)	Machine Height (m)	Machine Surface Temperature (℃)	5μm Stratification Height (m)
$A_1B_1C_1D_1$	0.083/1	22	1.5	32	1.66
$A_1B_2C_2D_2$	0.083/1	26	2.0	35	2.42
$A_1B_3C_3D_3$	0.083/1	30	3.0	37	3.89
$A_2B_1C_2D_3$	0.250/3	22	2.0	37	3.76
$A_2B_2C_3D_1$	0.250/3	26	3.0	32	3.55
$A_2B_3C_1D_2$	0.250/3	30	1.5	35	3.41
$A_3B_1C_3D_2$	0.500/6	22	3.0	35	4.13
$A_3B_2C_1D_3$	0.500/6	26	1.5	37	3.47
$A_3B_3C_2D_1$	0.500/6	30	2.0	32	3.47
K1	7.98	9.55	8.55	8.68	-
K2	10.72	9.44	9.65	9.96	-
K3	11.07	10.77	11.57	11.12	-
$\overline{K1}$	2.66	3.18	2.85	2.89	-
$\overline{K2}$	3.57	3.15	3.22	3.32	-
$\overline{K3}$	3.69	3.59	3.86	3.71	-
Range	1.03	0.44	1.01	0.81	-
sort	A>C>D>B				

Table S8. Orthogonal analysis table of workspace inhomogeneity factor for particles of size 5μm

Orthogonal Code	Supply Air Velocity ($m \cdot s^{-1}$) /Air Change Rate (ACR)	Supply Air Temperature (℃)	Machine Height (m)	Machine Surface Temperature (℃)	5μm Inhomogeneity Factor of Workspace
$A_1B_1C_1D_1$	0.083/1	22	1.5	32	0.85
$A_1B_2C_2D_2$	0.083/1	26	2.0	35	0.65
$A_1B_3C_3D_3$	0.083/1	30	3.0	37	0.20
$A_2B_1C_2D_3$	0.250/3	22	2.0	37	0.14
$A_2B_2C_3D_1$	0.250/3	26	3.0	32	0.22
$A_2B_3C_1D_2$	0.250/3	30	1.5	35	0.29
$A_3B_1C_3D_2$	0.500/6	22	3.0	35	0.11
$A_3B_2C_1D_3$	0.500/6	26	1.5	37	0.35
$A_3B_3C_2D_1$	0.500/6	30	2.0	32	0.41
K1	1.70	1.09	1.49	1.48	-
K2	0.64	1.21	1.20	1.04	-
K3	0.87	0.90	0.52	0.68	-
$\overline{K1}$	0.57	0.36	0.50	0.49	-
$\overline{K2}$	0.21	0.40	0.40	0.35	-
$\overline{K3}$	0.29	0.30	0.17	0.23	-
Range	0.35	0.10	0.32	0.27	-
sort	A>C>D>B				

Table S9. Orthogonal analysis table of stratification height for particles of size 10μm

Orthogonal Code	Supply Air Velocity ($m \cdot s^{-1}$) /Air Change Rate (ACR)	Supply Air Temperature (℃)	Machine Height (m)	Machine Surface Temperature (℃)	10μm Stratification Height (m)
$A_1B_1C_1D_1$	0.083/1	22	1.5	32	0.88
$A_1B_2C_2D_2$	0.083/1	26	2.0	35	1.42
$A_1B_3C_3D_3$	0.083/1	30	3.0	37	3.12
$A_2B_1C_2D_3$	0.250/3	22	2.0	37	3.02
$A_2B_2C_3D_1$	0.250/3	26	3.0	32	2.90
$A_2B_3C_1D_2$	0.250/3	30	1.5	35	2.86
$A_3B_1C_3D_2$	0.500/6	22	3.0	35	3.71
$A_3B_2C_1D_3$	0.500/6	26	1.5	37	3.15
$A_3B_3C_2D_1$	0.500/6	30	2.0	32	3.25
K1	5.42	7.61	6.89	7.03	-
K2	8.78	7.47	7.68	7.98	-
K3	10.11	9.23	9.73	9.29	-
$\overline{K1}$	1.81	2.54	2.30	2.34	-
$\overline{K2}$	2.93	2.49	2.56	2.66	-
$\overline{K3}$	3.37	3.08	3.24	3.10	-
Range	1.56	0.59	0.95	0.75	-
sort	A>C>D>B				

Table S10. Orthogonal analysis table of workspace inhomogeneity factor for particles of size 10μm

Horizontal coding	Rate of ventilation (ACR)	Air supply temperature (℃)	Machine tool height (m)	Machine tool temperature (℃)	10μm Non-uniformity coefficient of working area (m)
$A_1B_1C_1D_1$	1	22	1.5	32	0.77
$A_1B_2C_2D_2$	1	26	2.0	35	0.81
$A_1B_3C_3D_3$	1	30	3.0	37	0.59
$A_2B_1C_2D_3$	3	22	2.0	37	0.64
$A_2B_2C_3D_1$	3	26	3.0	32	0.64
$A_2B_3C_1D_2$	3	30	1.5	35	0.69
$A_3B_1C_3D_2$	6	22	3.0	35	0.22
$A_3B_2C_1D_3$	6	26	1.5	37	0.51
$A_3B_3C_2D_1$	6	30	2.0	32	0.51
K1	2.17	1.63	1.98	1.92	-
K2	1.98	1.97	1.96	1.73	-
K3	1.25	1.80	1.46	1.74	-
$\overline{K1}$	0.72	0.54	0.66	0.64	-
$\overline{K2}$	0.66	0.66	0.65	0.58	-
$\overline{K3}$	0.42	0.60	0.49	0.58	-
Range	0.31	0.11	0.17	0.06	-
sort	A>C>D>B				
